# Investigating the gut microbiota's influence on psoriasis and psoriatic arthritis risk: a Mendelian randomization analysis

**DOI:** 10.1093/pcmedi/pbad023

**Published:** 2023-09-15

**Authors:** Nianzhou Yu, Jiayi Wang, Yuancheng Liu, Yeye Guo

**Affiliations:** Department of Dermatology, Hunan Engineering Research Center of Skin Health and Disease, Hunan Key Laboratory of Skin Cancer and Psoriasis, Xiangya Hospital, Central South University, Changsha 410008, China; National Clinical Research Center for Geriatric Disorders, Xiangya Hospital, Central South University, Changsha 410008, China; Xiangya School of Medicine, Central South University, Changsha 410083, China; Department of Dermatology, Hunan Engineering Research Center of Skin Health and Disease, Hunan Key Laboratory of Skin Cancer and Psoriasis, Xiangya Hospital, Central South University, Changsha 410008, China; National Clinical Research Center for Geriatric Disorders, Xiangya Hospital, Central South University, Changsha 410008, China; Department of Dermatology, Hunan Engineering Research Center of Skin Health and Disease, Hunan Key Laboratory of Skin Cancer and Psoriasis, Xiangya Hospital, Central South University, Changsha 410008, China; National Clinical Research Center for Geriatric Disorders, Xiangya Hospital, Central South University, Changsha 410008, China

**Keywords:** gut microbiota, psoriasis, psoriatic arthritis, genetic, Mendelian randomization, causal effect

## Abstract

**Background:**

Numerous investigations have revealed the interplay between gut microbiota (GM) and psoriasis (Ps) and psoriatic arthritis (PsA). However, the causal relationship between them remains unknown.

**Methods:**

We curated a collection of genetic variants (*P* < 1 × 10^−5^) associated with GM (*n* = 18 340) derived from the MiBioGen study. To explore the intricate relationship between GM and Ps as well as PsA, we harnessed the comprehensive resources of the FinnGen database, encompassing a vast cohort of individuals, including 4510 Ps cases and 212 242 controls and 1637 PsA cases and 212 242 controls. Mendelian randomization (MR) was used, including an inverse variance weighting method, followed by a sensitivity analysis to verify the robustness of the results.

**Results:**

For Ps, some bacterial taxa, including *Lactococcus, Ruminiclostridium 5*, and *Eubacterium fissicatena*, were identified as risk factors; but *Odoribacter* demonstrated a protective effect against Ps. In the case of PsA, *Lactococcus, Verrucomicrobiales, Akkermansia, Coprococcus 1*, and *Verrucomicrobiaceae* were identified as risk factors; *Odoribacter* and *Rikenellaceae* exhibited a protective effect against the development of PsA.

**Conclusion:**

Our study establishes a causal link between the GM and Ps and PsA. These findings provide insights into the underlying mechanisms and suggest potential therapeutic targets.

## Introduction

Psoriasis (Ps), a chronic inflammatory skin disorder, is characterized by aberrant proliferation of keratinocytes and infiltration of immune cells into the epidermis.^1^, ^2^ The condition affects a significant portion of the population, with varying prevalence among different ethnic groups. Psoriatic arthritis (PsA), a severe comorbidity of Ps, manifests as joint pain, swelling, and rigidity.^2^ Both Ps and PsA exhibit a strong genetic predisposition, with heritability estimates ranging from 60% to 100%.^1^, ^3^ Furthermore, the prevalence of these conditions is still on the rise.^3^

Ps and PsA have garnered significant research attention to investigate their intricate relationship and potential implications in disease pathogenesis, progression, and management.^4^ Observations have revealed distinct alterations in the gut microbiome (GM) of individuals with Ps and PsA, presenting a unique dysbiosis pattern.^5^, ^6^ Several hypotheses have been proposed to elucidate the role of the GM in the pathogenesis of Ps and PsA, encompassing factors such as intestinal permeability, perturbed immune homeostasis, and imbalances in specific bacteria producing short- and medium-chain fatty acids.^5^, ^6^ Notably, interventions aimed at restoring the microbiome have exhibited promise as preventive and therapeutic strategies for Ps and PsA.^7^ For instance, in murine models displaying Ps-like skin inflammation, oral administration of a broad-spectrum antibiotic effectively mitigated the severity of skin inflammation by downregulating the Th17 immune response.^8^ However, it is crucial to acknowledge that substantial heterogeneity exists among studies investigating alterations in gut microbial composition and Ps, necessitating unified methodological standards and large-scale investigations to comprehensively understand the microbiota's contribution to Ps pathogenesis and explore its potential as a therapeutic target.^5–7^

Mendelian randomization (MR) is a statistical method used in genetic epidemiology research to investigate causal relationships between exposures and outcomes by utilizing genetic variants as instrumental variables.^9^ MR analyses provide valuable insights into the potential causal relationships between the GM and various health conditions. By utilizing genetic variants as instrumental variables, researchers can explore the role of the GM in disease development and identify specific microbial taxa that may be causally linked to certain conditions. These findings contribute to a better understanding of the complex interactions between the GM and human health.

In this study, we conducted a two-sample MR investigation using large-scale genome-wide association study (GWAS) data of GM, Ps, and PsA. The objective of our study was to uncover the potential causal effects of 196 GM taxa on Ps and PsA.

## Materials and methods

### Data sources and filter instrumental variables

The study design framework is depicted in the graphical abstract, illustrating the structure of our investigation. The MiBioGen group conducted an extensive genome-wide meta-analysis of GM composition, incorporating genetic variation data pertaining to the gut microbiota.^10^ This remarkable research endeavor encompassed a cohort of 18 340 individuals hailing from diverse regions, such as the USA, the UK, Finland, Sweden, Denmark, The Netherlands, and other countries. The comprehensive dataset employed in this study encompassed 16S rRNA gene sequencing profiles and genotyping information. Through meticulous analysis, we identified and classified bacteria at various taxonomic levels, including 9 phyla, 16 classes, 20 orders, 35 families, and 131 genera. Subsequently, 3 unidentified families and 12 unknown genera were excluded from the dataset, resulting in the inclusion of 9 phyla, 16 classes, 20 orders, 32 families, and 119 genera for further analysis in the subsequent MR investigation (supplementary Table 1, see online supplementary material).

To ensure the selection of high-quality genetic data, we chose the GWAS dataset with the most comprehensive coverage of single nucleotide polymorphisms (SNPs) published in 2021, sourced from the FinnGen project.^11^ This specific GWAS dataset focused on the phenotypes “psoriasis” and “psoriatic arthritis” and incorporated Finnish adult subjects, consisting of 4510 cases and 212 242 controls for Ps and 1637 cases and 212 242 controls for PsA.

To investigate potential causal links and associations between GM and Ps and PsA, it is essential to select valid instrumental variables (IVs) that satisfy three key assumptions: (1) the correlation hypothesis, (2) the exclusivity hypothesis, and (3) the independence assumption.^9^ Due to the limited number of SNPs available for MR analysis, we set a lenient threshold of *P* < 1 × 10^−5^. To ensure the independence of each SNP, we applied a linkage disequilibrium (LD) factor (*r*^2^) of 0.01 and a clumping window width (kb) of 10 000.^12^ Subsequently, we extracted information on SNPs associated with the aforementioned intestinal flora from the summary GWAS data on Ps and PsA. We eliminated missing SNPs and set the minor allele frequency (MAF) at 0.01.^13^ Additionally, we excluded all SNPs with palindromic structures to mitigate the influence of alleles on the results. To examine the presence of bias in the causal relationship between intestinal flora and Ps and PsA due to weak IVs, we employed the *F* value. When the *F*-statistic was <10, we considered the used SNP a weak IV and excluded it from the analysis.^14^ To evaluate the potential influence of confounding factors, we utilized the PhenoScanner V2 online tool^15^ and referred to the European Dermatological Association Guidelines on Ps and PsA.^16^ SNPs that showed associations with known confounders were subsequently excluded from the analysis.

### MR analysis

In this study, we utilized four different approaches, namely, MR-Egger, weighted median, random-effect inverse variance weighted (IVW), and weighted mode, to perform the MR analysis and calculate causal estimates between GM composition and the risk of Ps and PsA. Each approach has its specific requirements and assumptions.^17^ In the MR analysis conducted in this study, we adopted the IVW method as the cornerstone of the analytical framework for probing the association among bacterial taxa, Ps, and PsA. The IVW method, a widely embraced tool, serves as a robust means to derive causal estimates for discrete variables.^18^ By employing these approaches, the study aimed to assess the potential causal effects of GM composition on Ps and PsA and provide insights into their relationship.

### Sensitivity analysis

The sensitivity analysis encompassed a heterogeneity test and a multiplicity of validity test. To confirm IV heterogeneity, Cochran's Q-test was employed, and a *P* value < 0.05 was considered indicative of the absence of heterogeneity.^19^ To ensure that the selected IV does not affect the outcome variable of Ps and PsA risk through biological pathways other than the gut microbiota, we conducted an evaluation of the pleiotropic associations between the IV and other potential confounding factors utilizing the MR-Egger intercept test.^17^ In this regression methodology, the slope assumes the role of an estimate for the causal effect, while the intercept signifies the mean level of the genetic variant, serving as a metric for the assessment of pleiotropic effects.^20^ MR-PRESSO also aggregated the residuals for each SNP to evaluate the magnitude of horizontal pleiotropy. The MR-PRESSO outlier test facilitated the identification of outlier SNPs that contributed to pleiotropy at the overall level.^21^ The impact of individual outliers on the overall results was assessed using a leave-one-out analysis, calculating the remaining SNP effects after iteratively removing each SNP. The MR-Egger intercept test, MR-PRESSO, and leave-one-out analysis methods were employed to identify and eliminate SNPs exhibiting pleiotropy or heterogeneity.^21^ Furthermore, the causal direction was investigated using the MR Steiger test.^22^ Additionally, a reverse MR analysis was performed. The MR analyses were conducted using the R (version 4.3.0) computational environment, utilizing the “TwoSampleMR” and “MR-PRESSO” packages. The R package ‘forestploter’ was employed to generate some figures. Statistical significance for causal effects was determined using a *P* value threshold of <0.05.

## Results

By applying genome-wide significance threshold screening (*P* < 1 × 10^−5^), LD tests, harmonization, and verification of *F*-statistics, multiple SNPs were identified as IVs for each of the 196 bacterial taxa. The *F*-statistics of all the retained SNPs demonstrate a correlation strength >10, indicating a sufficient association between the instrumental variables and their corresponding bacterial taxa (supplementary Tables S2 and S3, see online supplementary material). Consequently, our study had negligible instrument bias.

### Effect of gut microbiome on psoriasis and psoriatic arthritis

**Figure 1.**
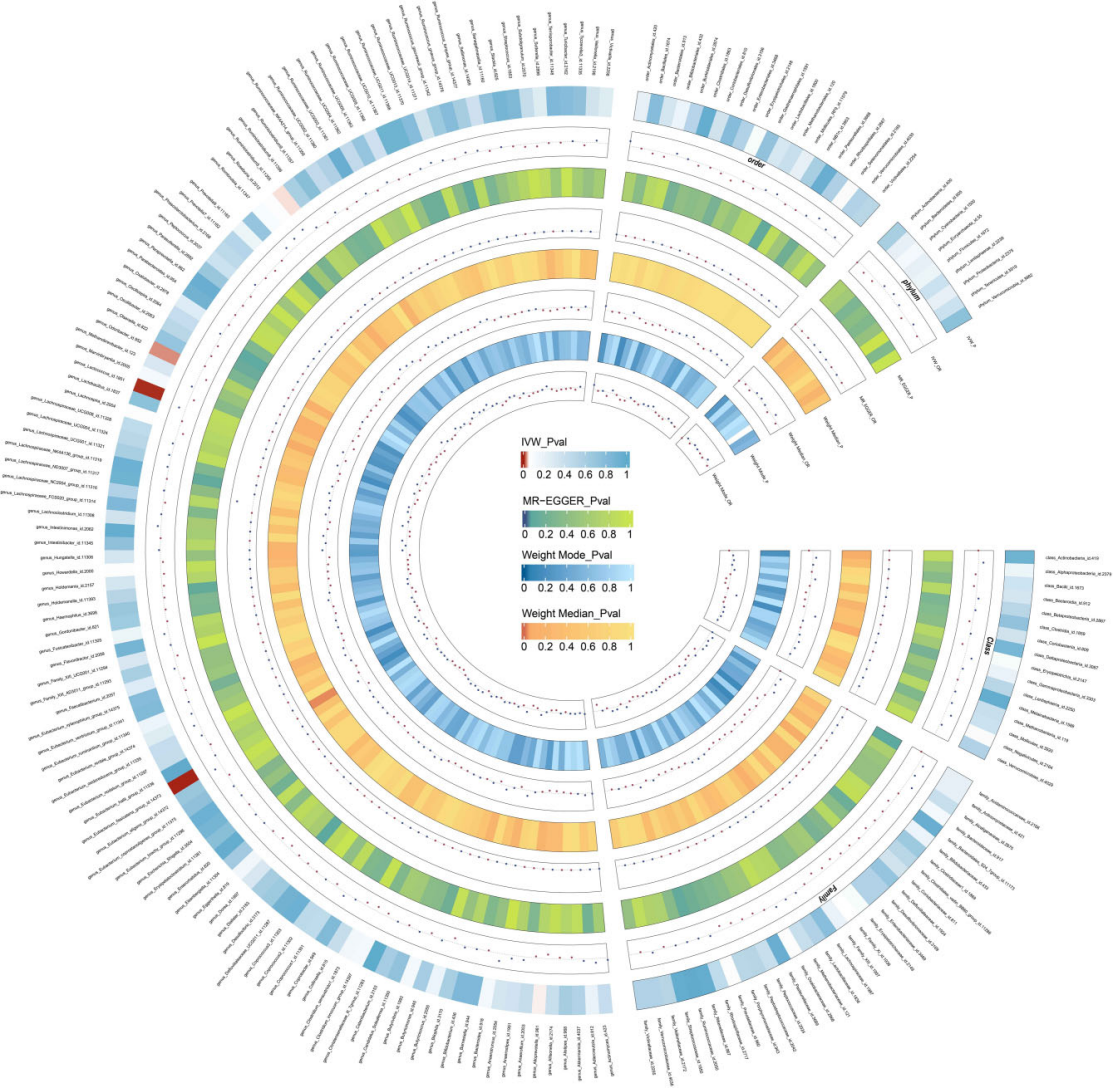
Circus plot showing analysis results of all gut microbiota on Ps. The circular representation depicts the estimates obtained through the IVW, weighted media, and MR-Egger methods, moving from the outer to the inner circle. The classification of GM was based on order, phylum, class, family, and genus. The varying shades of color in the circle represent the magnitude of the *P* values, with the corresponding label inside the circle. Ps, Psoriasis; MR, Mendelian randomization; IVW, inverse variance-weighted; WM, weighted median. *P* < 0.05.

**Figure 2.**
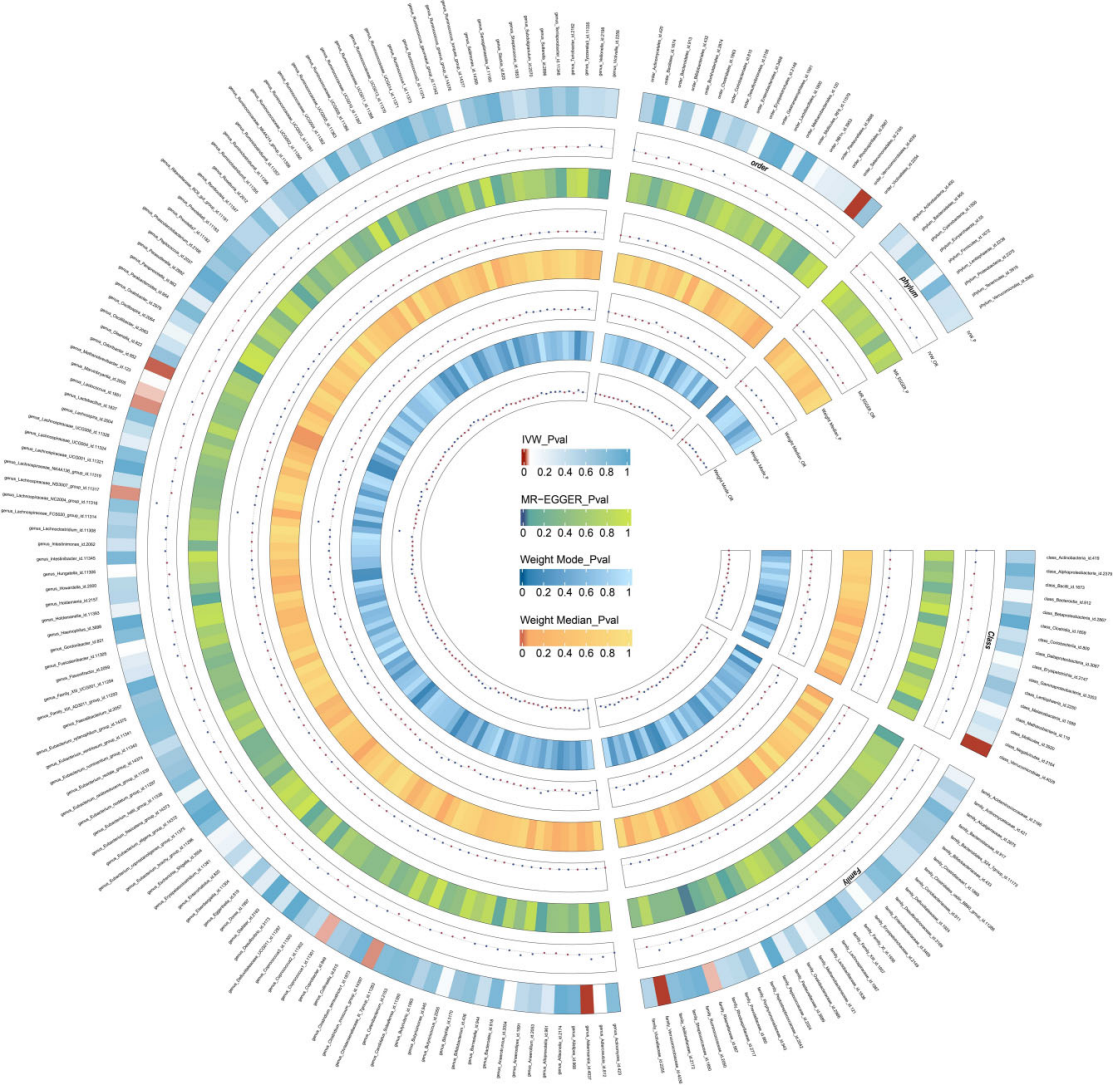
Circus plot showing analysis results of all gut microbiota on PsA. The circular representation depicts the estimates obtained through the IVW, weighted media, and MR-Egger methods, moving from the outer to the inner circle. The classification of GM was based on order, phylum, class, family, and genus. The varying shades of color in the circle represent the magnitude of the *P* values, with the corresponding label inside the circle. PsA, Psoriatic arthritis; MR, Mendelian randomization; IVW, inverse variance-weighted; WM, weighted median. *P* < 0.05.

**Figure 3.**
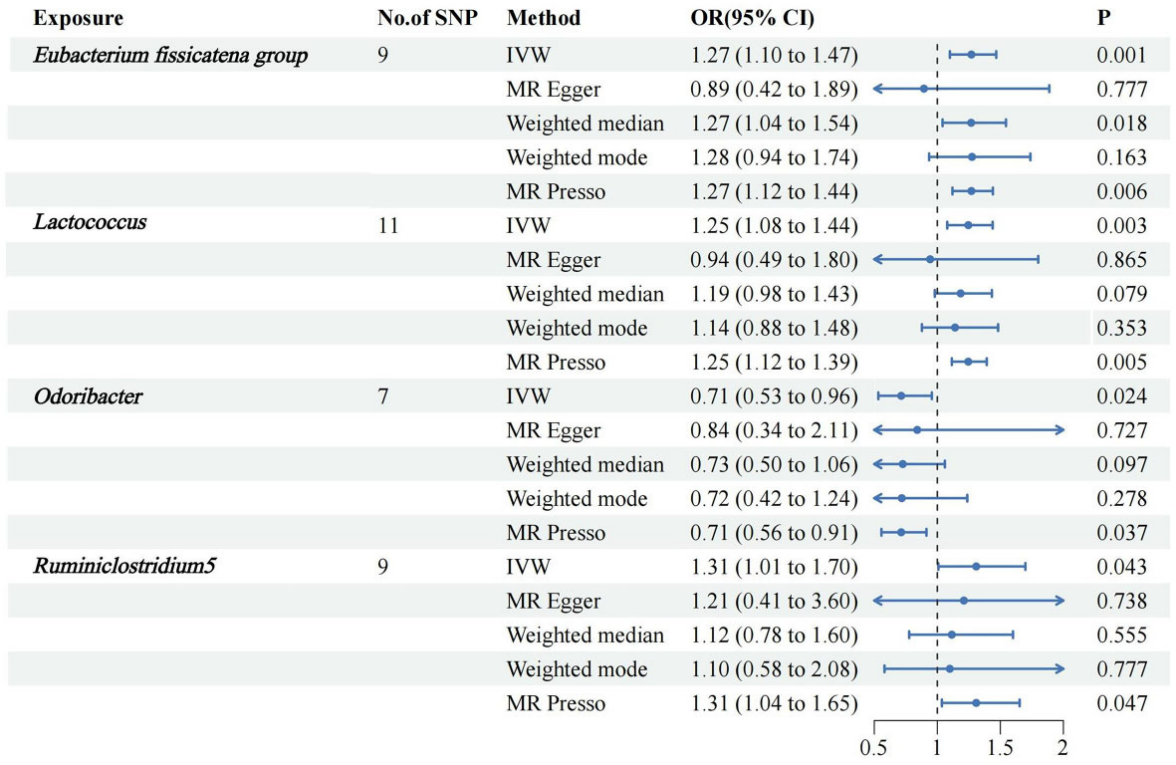
Forest plots for the association of GM and genetic susceptibility to Ps, analysed with MR. Ps, Psoriasis; GM, gut microbiota; OR, odds ratio; CI, confidence interval. *P* < 0.05.

**Figure 4.**
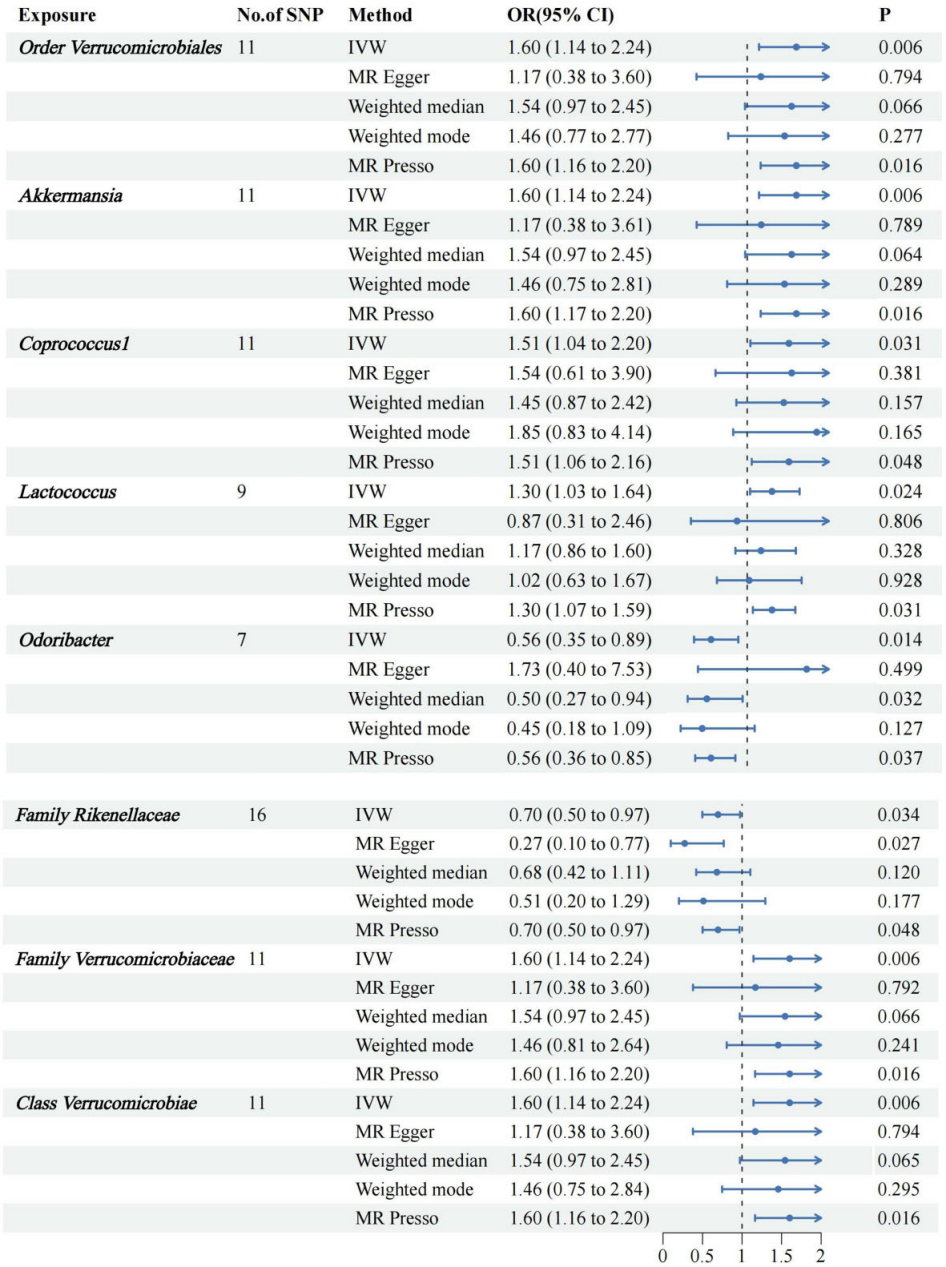
Forest plots for the association of GM and genetic susceptibility to PsA, analysed with MR. PsA, Psoriatic arthritis, GM, gut microbiota, OR, odds ratio; CI, confidence interval. *P* < 0.05.

We screened 2033 SNPs as instrumental variables from 196 gut microbiota. The results of the MR analysis for IVs are shown in circus plots (Figs. 1 and 2) and detailed in supplementary Tables S4 and S5 (see online supplementary material). Our study demonstrated the presence of 8 taxa of GM that accelerate the onset of Ps and PsA, while 2 taxa of GM have a protective effect, reducing the risk of these conditions. In Ps, the results of IVW indicated suggestive causal effects of genetically predicted increased abundance of *Odoribacter* (OR, 0.71; 95% CI, 0.53–0.96; *P*  =  0.024), which exhibited protective effects against Ps risk (Fig. 3). Conversely, *Eubacterium fissicatena* at the group level [(odds ratio (OR), 1.14; 95% confidence interval (CI), 0.99–1.30; *P*  =  0.001), *Ruminiclostridium 5* (OR, 1.31; 95% CI, 1.01–1.70; *P*  =  0.043), and *Lactococcus* (OR, 1.25; 95% CI, 1.08–1.44; *P*  =  0.003) were associated with a higher risk of Ps. For PsA, the IVW analyses demonstrated suggestive causal effects of genetically predicted increased abundance of *Rikenellaceae* at the family level (OR, 0.70; 95% CI, 0.50–0.97; *P*  =  0.034), which exhibited protective effects against PsA risk. Conversely, *Verrucomicrobiales* at the order level (OR, 1.60; 95% CI, 1.14–1.24; *P*  =  0.006), *Coprococcus1* (OR, 1.51; 95% CI, 1.03–2.19; *P*  =  0.03), *Akkermansia* (OR, 1.60; 95% CI, 1.14–1.24; *P*  =  0.006), *Verrucomicrobiae* at the class level (OR, 1.60; 95% CI, 1.14–1.24; *P*  =  0.006), and *Verrucomicrobiaceae* at the family level (OR, 1.60; 95% CI, 1.14–1.24; *P*  =  0.006) were associated with a higher risk of PsA (Fig. 4). It is worth noting that the order *Verrucomicrobiales*, along with the families *Verrucomicrobiaceae* and *Rikenellaceae*, belongs to the subclass *Verrucomicrobiae*. As a result, there may be significant overlap in the SNPs present in these four sets, as detailed in supplementary Table S3. Figures 3 and 4 show the results of the four analytical methods (MR-Egger, weighted median, IVW, and weighted mode), including *P* values, ORs and 95% CIs.

### Sensitivity analysis

The forest plots showed that several IV intercepts in the MR-Egger analysis deviated from zero. Furthermore, several IVs in the MR leave-one-out sensitivity analysis had effect values that span zero within the 95% CI, suggesting potential instability in the findings (supplementary Figs. S1–S4, see online supplementary material). However, further statistical analysis revealed that all *P* values indicating heterogeneity among the bacterial taxa mentioned above were >0.05 (supplementary Tables S6 and S7, see online supplementary material). MR-Egger's test found no evidence of horizontal pleiotropy, indicating that the genetic variants used as instruments for MR did not have pleiotropic effects (supplementary Tables S4 and S5). Additionally, the reliability of the results was assessed using MR-PRESSO, which identified no outliers; and the MR Presso Global Test results all exceeded the threshold of 0.05 (supplementary Tables S6 and S7). We recognized that overly stringent filtering criteria may inadvertently exclude some valid positive findings. Hence, we continued to affirm the causal relationships of the aforementioned microbial taxa as valid.

### Reverse MR analysis

To mitigate the influence of reverse causality on the aforementioned findings, we conducted a reverse MR analysis with significant gut flora as the outcome and Ps and PsA as the exposure variables. Our analysis did not provide any additional evidence supporting a causal effect of Ps and PsA (supplementary Figs. S5 and S6, see online supplementary material).

## Discussion

Ps, a chronic autoimmune disease characterized by arthritis, often leads to the development of PsA as a common complication. PsA is estimated to occur in 7% to 42% of individuals with Ps, and its prevalence gradually increases as the duration of Ps persists.^23^ Previous studies have suggested a potential association between the GM and the development of Ps and PsA.^5^ However, direct evidence establishing a causal correlation is currently lacking. In this study, we conducted a comprehensive investigation using MR analysis to explore the relationship between 196 gut bacterial taxa and the occurrence of Ps and PsA. Regarding Ps, we found that certain bacterial taxa, such as *Lactococcus, Ruminiclostridium 5*, and *E. fissicatena*, were identified as risk factors. Conversely, *Odoribacter* demonstrated a protective effect against Ps. In the case of PsA, our results revealed a distinct set of risk factors and protective factors among gut bacterial taxa. *Lactococcus, Verrucomicrobiales, Akkermansia, Coprococcus 1*, and *Verrucomicrobiaceae* were identified as risk factors for PsA. On the other hand, *Odoribacter* and *Rikenellaceae* exhibited a protective effect against the development of PsA.

Interestingly, among these risk factors, only one species of *Lactococcus* was shared between Ps and PsA, while the remaining bacteria differed. These findings suggest that while PsA is a complication of Ps, its pathogenesis does not completely align with that of Ps. Several of the risky bacteria identified in our study align with previous research findings. For instance, the abundance of *Lactococcus* has been shown to increase in the GM of patients with Ps.^24^, ^25^*Ruminiclostridium 5*, another risky bacterium, exhibited increased abundance in an experimental model of Ps induced by imiquimod.^26^*Odoribacter*, a coprotective bacterium for both Ps and PsA, has been reported to be more abundant in healthy individuals than in patients.^27^ Furthermore, studies have demonstrated that the compound resveratrol can increase the abundance of *Odoribacter* groups, thereby restoring intestinal ecology in mice.^28^ Interestingly, both oral and topical administration of resveratrol have shown potential in alleviating imiquimod-induced Ps-like dermatitis,^29^, ^30^ suggesting that *Odoribacter* might serve as a probiotic for Ps.

Additionally, among the protective flora associated with PsA, *Rikenellaceae* has been found to decrease in abundance in the GM of Ps patients.^31^ This finding underscores the shared characteristics and differences between Ps and PsA. The results concerning *Verrucomicrobiae, Akkermansia*, and *Coprococcus* in our study further support this notion. Previous reports have shown a decreased abundance of these bacterial types in the intestinal flora of Ps patients,^32–35^ indicating their potential as protective factors against Ps. However, our findings indicate that they act as risky flora in PsA, further emphasizing the distinct disease characteristics and pathogenesis of PsA compared to Ps.

Currently, there is a prevailing belief that Ps and PsA share common pathogenic factors, including genetic risk alleles, environmental triggers, and cytokine pathways. However, it is important to note that the resident cells in the skin and joints differ significantly, and the clinical manifestations of musculoskeletal disorders and skin lesions exhibit substantial variation among individuals.^33^, ^36^, ^37^ Despite the common involvement of tumor necrosis factor (TNF) and the interleukin (IL)-23–IL-17 axis in the pathogenesis of both Ps and PsA, monotherapy targeting IL-17 or IL-23 has demonstrated high efficacy in Ps but not in PsA. Although the effectiveness in PsA is less pronounced, these observations further underscore the distinct pathogenic mechanisms underlying skin and joint diseases. One potential mechanism contributing to these differences lies in the microbiome and mucosal immunity. Studies have reported significant dysregulation of intestinal mucosal immune function in PsA patients.^38–40^ Moreover, compared to Ps patients, PsA patients exhibit lower overall intestinal diversity,^35^ suggesting that alterations in intestinal immune dynamics may contribute to synovial enthesis inflammation. Notably, a specific subset of osteoclast precursors, CD14^+^CD16^+^, has been identified in PsA patients but not in Ps patients.^41^ Typically, patients with Ps experience skin lesions first, followed by the onset of PsA. However, it is worth noting that ∼15% of cases present with arthritis and skin lesions occurring simultaneously or with arthritis preceding the skin lesions.^42^ A recent study revealed that patients who develop PsA as the initial symptom often delay seeking medical attention and initiating treatment, which can significantly impact long-term prognosis. Previous research has identified potential predictors of Ps progression to PsA. For instance, C-X-C motif chemokine ligand 10 (CXCL10) has been proposed as a predictive marker for the development of PsA.^43^ Another case‒control study demonstrated independent associations between PsA and serum levels of integrin beta 5 (ITGB5), Mac-2 binding protein (M2BP), and C-reactive protein (CRP).^44^ Furthermore, evaluation of the skin proteome and serum samples has revealed the presence of ITGB5 and periostin in PsA patients, distinguishing them from those with Ps alone.^45^ Our study suggests that specific gut flora analysis may aid in the early diagnosis of PsA among patients presenting with joint inflammation. Moreover, targeting shared pathogenic bacteria, such as *Lactococcus*, or considering probiotic supplementation with *Odoribacter* could potentially serve as treatment options for individuals with Ps and concomitant PsA.

In this study, we conducted a comprehensive investigation to explore the causal relationship between GM and Ps and PsA, utilizing publicly available GWAS summary statistics. We uncovered specific bacterial groups that hold the potential to influence the development of Ps and PsA. Certain intestinal flora were implicated in the pathogenesis of PsA, suggesting their potential role as early diagnostic indicators. Furthermore, we identified several GMs that exhibit a potential protective effect against the occurrence of Ps and PsA. These discoveries lay a solid foundation for future endeavors in the prevention and treatment of these conditions. One of the key strengths of our study lies in the rigorous utilization of the MR method, which effectively mitigates the impact of reverse causal associations and confounding factors. This methodological approach adds considerable robustness to our findings and enhances the validity of our causal inferences. Notably, our MR study encompassed a remarkably broad population, leveraging publicly available data at a minimal cost. This extensive coverage not only enhances the generalizability of our results but also augments the practicality and persuasiveness of our findings when compared to conventional observational studies. Naturally, it is essential to acknowledge the limitations of our research. First, the MiBioGen study includes gut microbiota GWAS data from various countries, including the USA and Europe, while FinnGen focuses solely on the Finnish population. Although MR analysis can combine individual-level and summary data,^46^ we should interpret our results cautiously due to potential genetic variation across these populations. Moreover, the causal relationships identified in our MR study rely on IVs meeting a rigorous genome-wide significance threshold (*P* < 1 × 10^−5^). It is crucial to recognize that this stringent criterion could potentially affect the precision of our results. Second, inconsistencies were observed in the analysis of certain bacterial populations, which could potentially be attributed to the utilization of MR-Egger's method for estimating causality. It is plausible that this method introduces bias by altering the Type 1 error rate, leading to inflated rates of Type 1 errors and subsequently influencing the OR.^21^ Third, we focused exclusively on the European population, which restricts the generalizability of our findings to other ethnicities or regions. Therefore, caution should be exercised in extrapolating our results to populations beyond the scope of our study. Moreover, we emphasize the importance of conducting further observational studies and laboratory-based investigations to validate and expand upon our current findings. By consolidating evidence from multiple research approaches, we can advance the knowledge base and provide a more robust understanding of the intricate relationship between the GM and the development of Ps and PsA.

## Supplementary Material

pbad023_Supplemental_Files
