# Supplementary material for: Investigating the gut microbiota's influence on psoriasis and psoriatic arthritis risk: a Mendelian randomization analysis
Source: Precis Clin Med. 2023 Sep 15;6(3):pbad023. doi: 10.1093/pcmedi/pbad023 (PMC10680138; doi:10.1093/pcmedi/pbad023)
Supplement: pbad023_Supplemental_Files [file pbad023_supplemental_files.zip › Supplementary_Figures.docx]

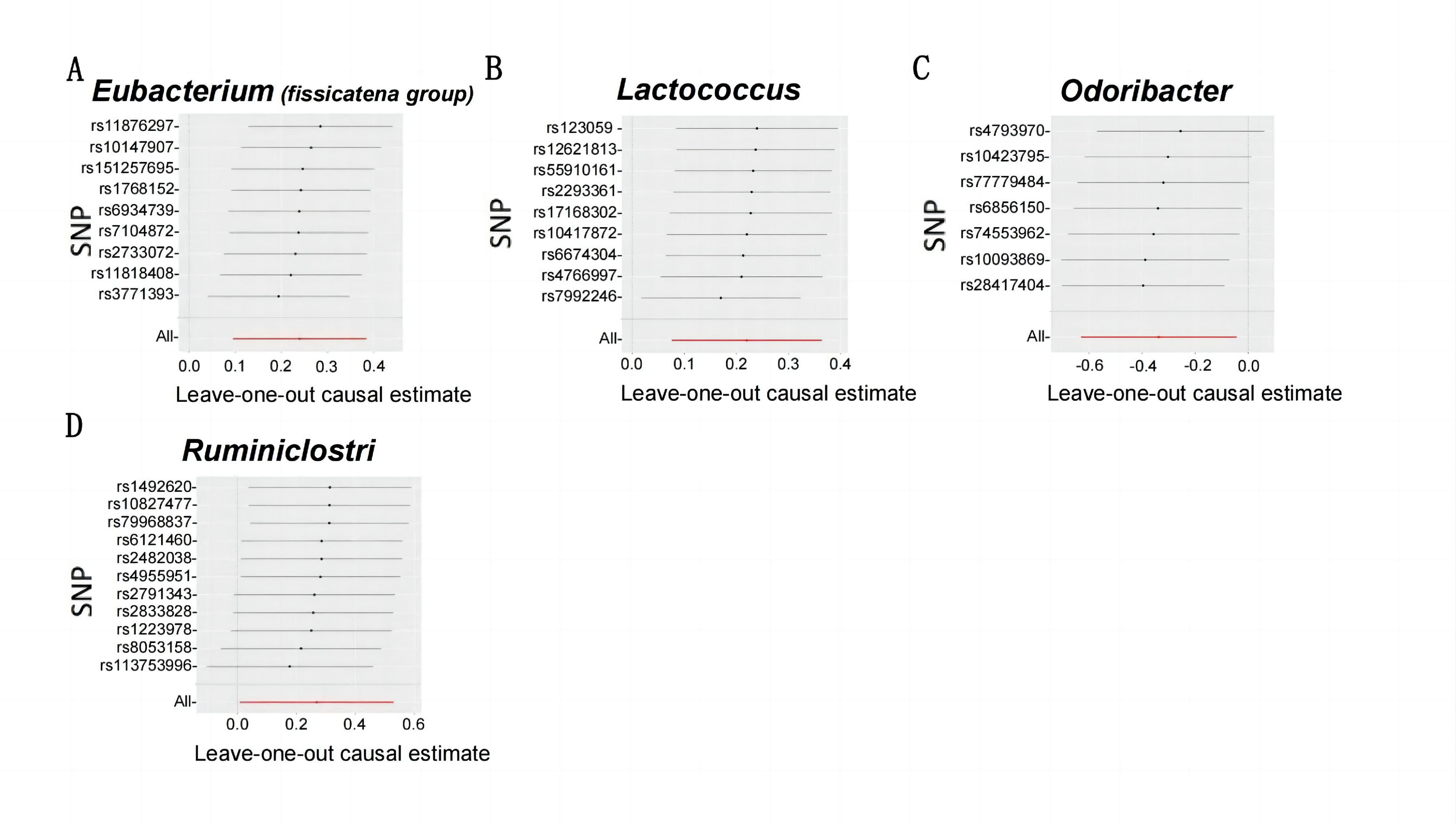
**Supplementary Figures**

**Figure S1.** MR leave-one-out sensitivity analysis for gut microbiota abundance on Ps. (A) Leave-one-out sensitivity analysis of the effect of Alloprevotella on Ps; (B) Leave-one-out sensitivity analysis of the effect of Lactococcus on Ps; (C) Leave-one-out sensitivity analysis of the effect of Odoribacter on Ps; (D) Leave-one-out sensitivity analysis of the effect of Ruminiclostridium5 on Ps; (E) Leave-one-out sensitivity analysis of the effect of Eubacterium (fissicatena group) on Ps. Ps, Psriasis.

**
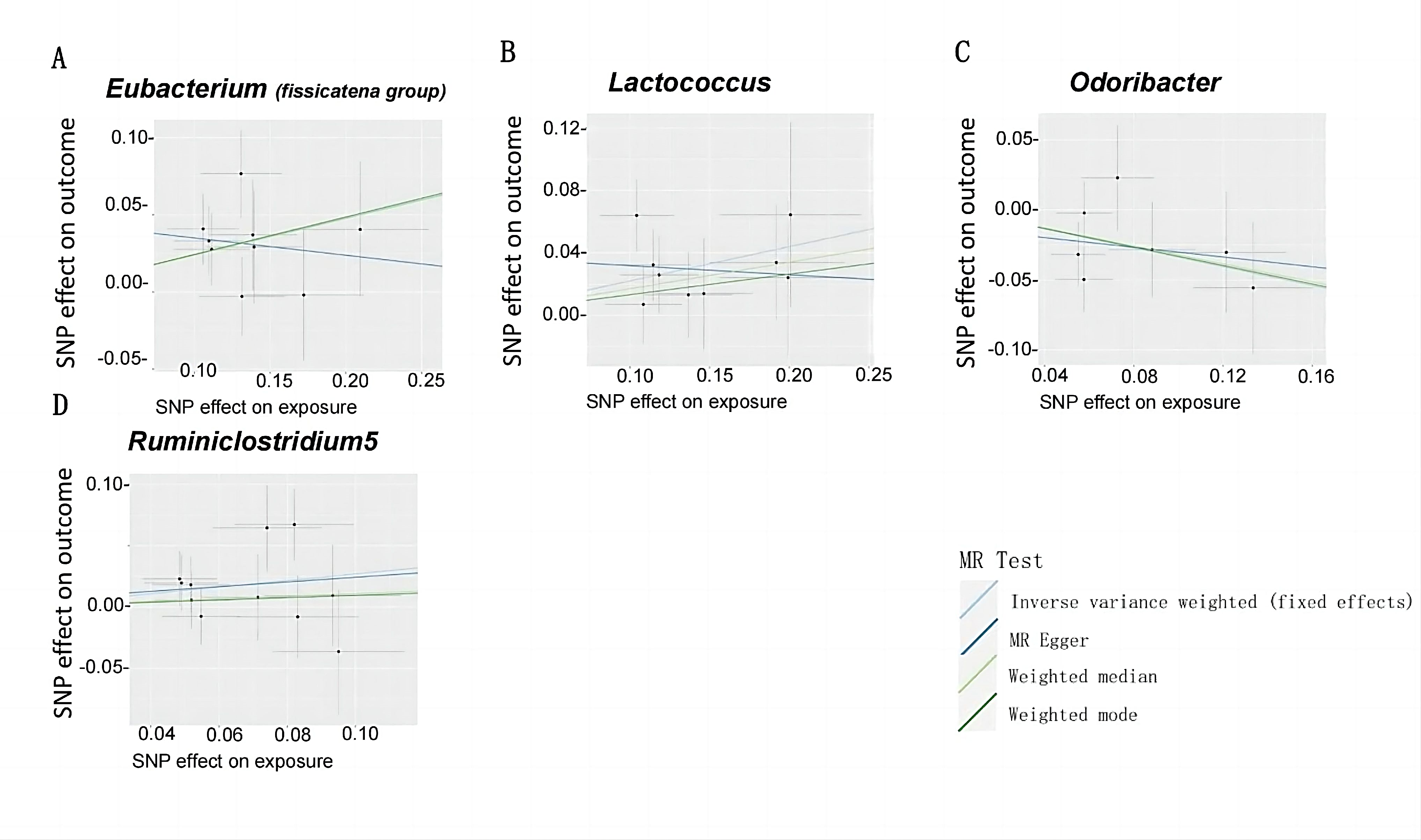
Figure S2.** Scatter plot of the 4 MR tests at the gut microbes. SNP effects were plotted as lines for the IVW test (light blue line), MR‒Egger regression (dark blue line), weighted median estimator (light green line), and weighted mode (dark green line). The slope of the line corresponds to the causal estimation. (A) Scatter plot of the effect of Eubacterium (Fissicatena group) on Ps. (B) Scatter plot of the effect of Lactococcus on Ps. (C) Scatter plot of the effect of Odoribacter on Ps. (D) Scatter plot of the effect of Ruminiclostridium5 on Ps. Ps, Psriasis.

**
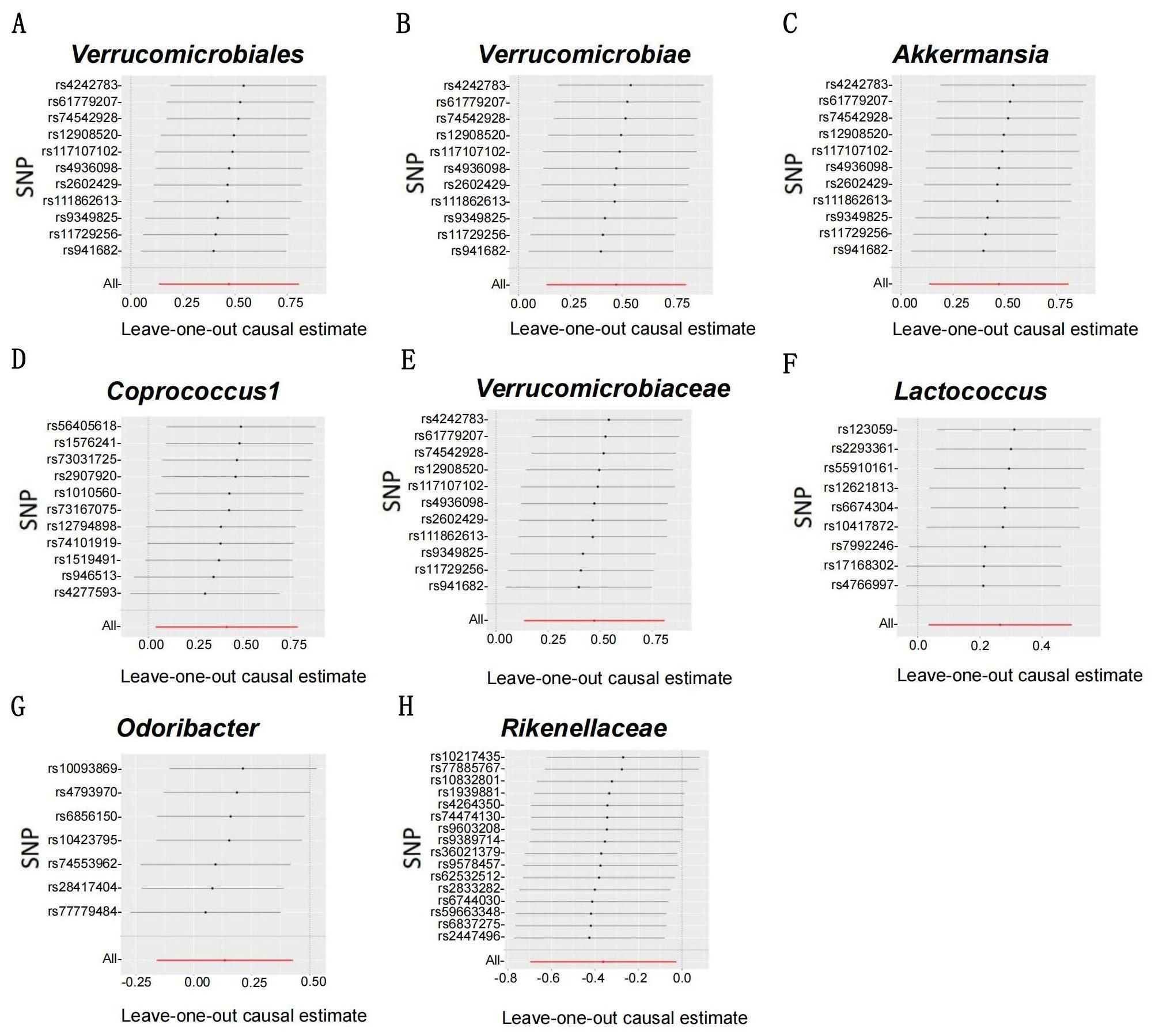
Figure S3.** MR leave-one-out sensitivity analysis for gut microbiota abundance on PsA. (A) Leave-one-out sensitivity analysis of the effect of Verrucomicrobiales on PsA; (B) Leave-one-out sensitivity analysis of the effect of Verrucomicrobiae on PsA; (C) Leave-one-out sensitivity analysis of the effect of Akkermansia on PsA; (D) Leave-one-out sensitivity analysis of the effect of Coprococcus1 on PsA; (E) Leave-one-out sensitivity analysis of the effect of Verrucomicrobieae on PsA; (F) Leave-one-out sensitivity analysis of the effect of Lactococcus on PsA; (G) Leave-one-out sensitivity analysis of the effect of Odoribacter on PsA; (H) Leave-one-out sensitivity analysis of the effect of Rikenellaceae on PsA. PsA, Psriatic arthritis; MR, Mendelian randomization; SNP, single-nucleotide polymorphism.

**
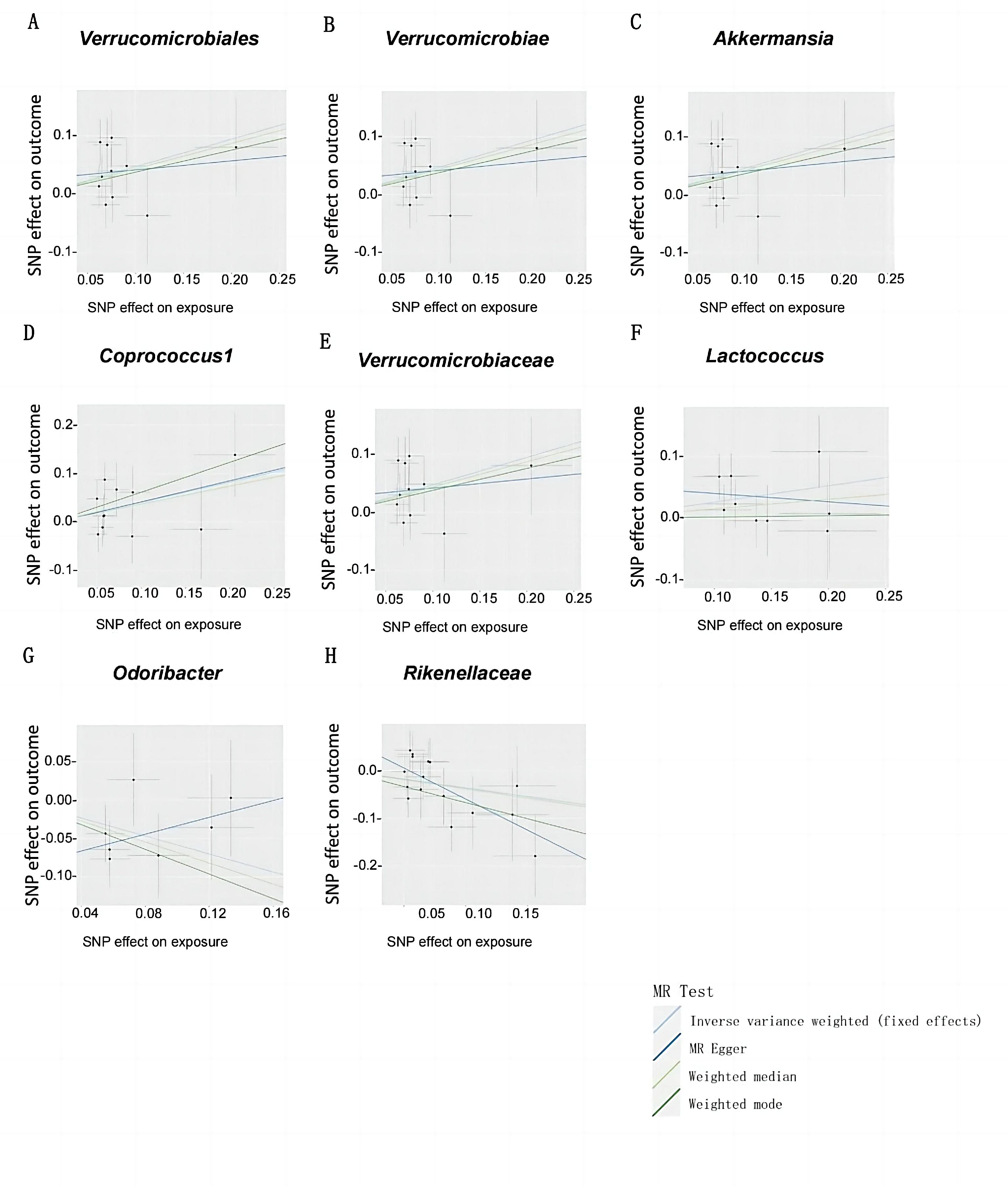
Figure S4.** Scatter plot of the 4 MR tests at the gut microbes. SNP effects were plotted as lines for the IVW test (light blue line), MR‒Egger regression (dark blue line), weighted median estimator (light green line), and weighted mode (dark green line). The slope of the line corresponds to the causal estimation. (A) Scatter plot of the effect of Verrucomicrobiales on PsA. (B) Scatter plot of the effect of Verrucomicrobiae on PsA. (C) Scatter plot of the effect of Akkermansia on PsA. (D) Scatter plot of the effect of Coprococcus1 on PsA. (E) Scatter plot of the effect of Verrucomicrobieae on PsA. (F) Scatter plot of the effect of Lactococcus on PsA. (G) Scatter plot of the effect of Odoribacter on PsA. (H) Scatter plot of the effect of Rikenellaceae on PsA.

**
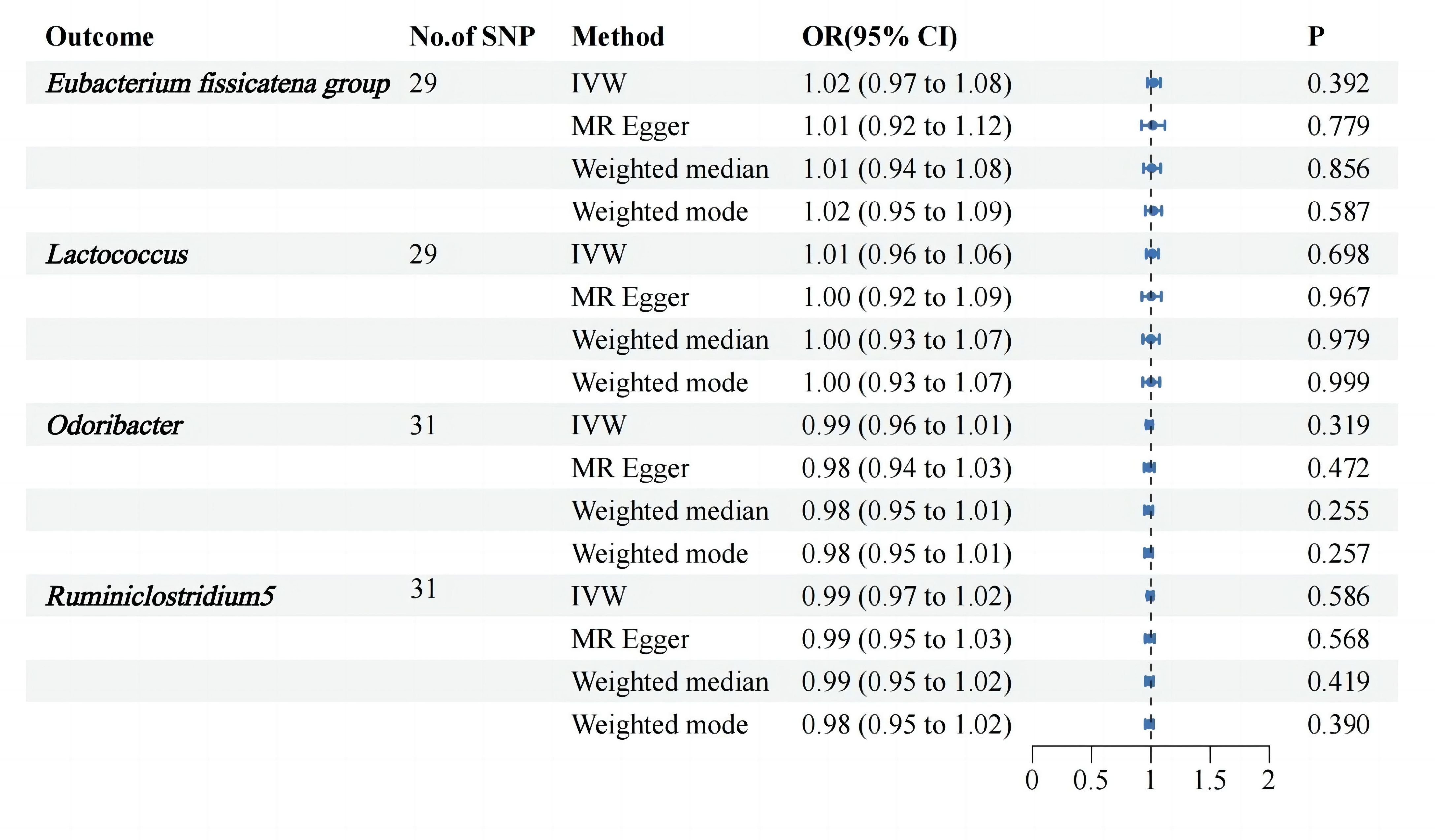
**

**Figure S5. Forest plots for the associations of genetic susceptibility to Ps with different Mendelian randomizations of positive GM.** Ps, psoriasis; GM, gut microbiota; OR, odds ratio; CI, confidence interval. *P* < 0.05.

**
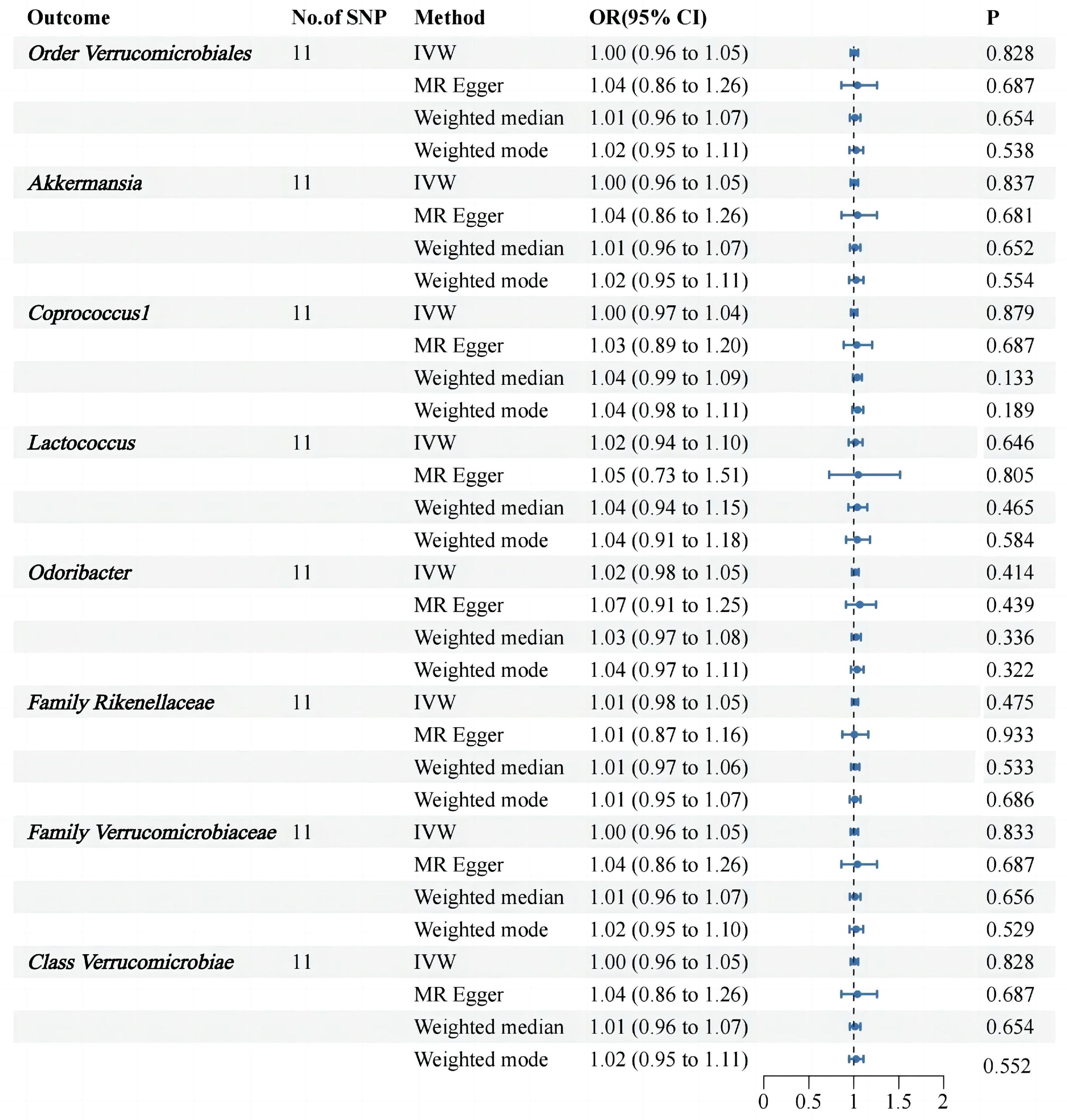
**

**Figure S6. Forest plots for the associations of genetic susceptibility to PsA with different Mendelian randomizations of positive GM.** PsA, psoriatic arthritis; GM, gut microbiota; OR, odds ratio; CI, confidence interval. *P* < 0.05.
